# Supplementary material for: Dopamine D1-like receptors modulate synchronized oscillations in the hippocampal–prefrontal–amygdala circuit in contextual fear
Source: Sci Rep. 2023 Oct 17;13:17631. doi: 10.1038/s41598-023-44772-6 (PMC10582086; doi:10.1038/s41598-023-44772-6)
Supplement: Supplementary file 1 — Supplementary Tables. [file 41598_2023_44772_MOESM1_ESM.docx]

**Supplementary Tables**

**Table S1.** Statistical analysis of acute SCH23390 effects on theta-gamma PAC before CFC (two-way ANOVA with repeated measures).

| **Within area** | | **F value** | **P value** |
| --- | --- | --- | --- |
| DH-DH | Main effect of treatment | F_(1,31)_=0.88 | P=0.36 |
|  | Main effect of frequency | F_(1,31)_=65.48 | P<0.0001 |
|  | Treatment x frequency interaction | F_(1,31)_=0.84 | P=0.37 |
| VH-VH | Main effect of treatment | F_(1,28)_=3.19 | P=0.085 |
|  | Main effect of frequency | F_(1,28)_=73.84 | P<0.0001 |
|  | Treatment x frequency interaction | F_(1,28)_=2.05 | P=0.16 |
| PL-PL | Main effect of treatment | F_(1,26)_=0.23 | P=0.64 |
|  | Main effect of frequency | F_(1,26)_=106.0 | P<0.0001 |
|  | Treatment x frequency interaction | F_(1,26)_=1.28 | P=0.27 |
| BLA-BLA | Main effect of treatment | F_(1,16)_=1.45 | P=0.25 |
|  | Main effect of frequency | F_(1,16)_=76.26 | P<0.0001 |
|  | Treatment x frequency interaction | F_(1,16)_=1.22 | P=0.29 |

| **Between areas** | | **F value** | **P value** |
| --- | --- | --- | --- |
| DH theta-  VH gamma | Main effect of treatment | F_(1,24)_=4.41 | P=0.046 |
|  | Main effect of frequency | F_(1,24)_=74.92 | P<0.0001 |
|  | Treatment x frequency interaction | F_(1,24)_=1.75 | P=0.20 |
| VH theta-  PL gamma | Main effect of treatment | F_(1,21)_=1.61 | P=0.22 |
|  | Main effect of frequency | F_(1,21)_=120.8 | P<0.0001 |
|  | Treatment x frequency interaction | F_(1,21)_=4.53 | P=0.045 |
| VH theta-BLA gamma | Main effect of treatment | F_(1,12)_=0.27 | P=0.61 |
|  | Main effect of frequency | F_(1,12)_=51.42 | P<0.0001 |
|  | Treatment x frequency interaction | F_(1,12)_=0.16 | P=0.69 |
| PL theta-BLA gamma | Main effect of treatment | F_(1,12)_=0.16 | P=0.70 |
|  | Main effect of frequency | F_(1,12)_=61.61 | P<0.0001 |
|  | Treatment x frequency interaction | F_(1,12)_=0.028 | P=0.87 |

**Table S2.** Statistical analysis of the effects of SCH23390 given before CFC on theta-gamma PAC at retrieval (two-way ANOVA with repeated measures).

| **Within area** | | **F value** | **P value** |
| --- | --- | --- | --- |
| DH-DH | Main effect of treatment | F_(1,35)_=0.17 | P=0.69 |
|  | Main effect of frequency | F_(1,35)_=64.96 | P<0.0001 |
|  | Treatment x frequency interaction | F_(1,35)_=0.34 | P=0.56 |
| VH-VH | Main effect of treatment | F_(1,30)_=3.08 | P=0.089 |
|  | Main effect of frequency | F_(1,30)_=37.85 | P<0.0001 |
|  | Treatment x frequency interaction | F_(1,30)_=0.82 | P=0.37 |
| PL-PL | Main effect of treatment | F_(1,28)_=0.45 | P=0.51 |
|  | Main effect of frequency | F_(1,28)_=6.30 | P=0.018 |
|  | Treatment x frequency interaction | F_(1,28)_=1.49 | P=0.23 |
| BLA-BLA | Main effect of treatment | F_(1,17)_=1.62 | P=0.22 |
|  | Main effect of frequency | F_(1,17)_=18.66 | P=0.0005 |
|  | Treatment x frequency interaction | F_(1,17)_=0.034 | P=0.86 |

| **Between areas** | | **F value** | **P value** |
| --- | --- | --- | --- |
| DH theta-  VH gamma | Main effect of treatment | F_(1,27)_=5.03 | P=0.03 |
|  | Main effect of frequency | F_(1,27)_=33.87 | P<0.0001 |
|  | Treatment x frequency interaction | F_(1,27)_=1.70 | P=0.20 |
| VH theta-  PL gamma | Main effect of treatment | F_(1,23)_=0.79 | P=0.38 |
|  | Main effect of frequency | F_(1,23)_=6.75 | P=0.016 |
|  | Treatment x frequency interaction | F_(1,23)_=0.66 | P=0.42 |
| VH theta-BLA gamma | Main effect of treatment | F_(1,14)_=1.28 | P=0.28 |
|  | Main effect of frequency | F_(1,14)_=20.40 | P=0.0005 |
|  | Treatment x frequency interaction | F_(1,14)_=0.71 | P=0.41 |
| PL theta-BLA gamma | Main effect of treatment | F_(1,13)_=1.64 | P=0.22 |
|  | Main effect of frequency | F_(1,13)_=22.39 | P=0.0004 |
|  | Treatment x frequency interaction | F_(1,13)_=0.85 | P=0.37 |

**Table S3.** Statistical analysis of movement effects on theta-gamma PAC at retrieval in the vehicle-treated controls (two-way ANOVA with repeated measures).

| **Within area** | | **F value** | **P value** |
| --- | --- | --- | --- |
| DH-DH | Main effect of locomotion | F_(1,25)_=0.040 | P=0.84 |
|  | Main effect of frequency | F_(1,25)_=33.68 | P<0.0001 |
|  | Locomotion x frequency interaction | F_(1,25)_=0.58 | P=0.45 |
| VH-VH | Main effect of locomotion | F_(1,21)_=0.10 | P=0.75 |
|  | Main effect of frequency | F_(1,21)_=12.83 | P=0.0018 |
|  | Locomotion x frequency interaction | F_(1,21)_=0.34 | P=0.57 |
| PL-PL | Main effect of locomotion | F_(1,16)_=1.04 | P=0.32 |
|  | Main effect of frequency | F_(1,16)_=10.43 | P=0.0052 |
|  | Locomotion x frequency interaction | F_(1,16)_=1.13 | P=0.30 |
| BLA-BLA | Main effect of locomotion | F_(1,7)_=4.05 | P=0.084 |
|  | Main effect of frequency | F_(1,7)_=23.63 | P=0.0018 |
|  | Locomotion x frequency interaction | F_(1,7)_=5.59 | P=0.050 |

| **Between areas** | | **F value** | **P value** |
| --- | --- | --- | --- |
| DH theta-  VH gamma | Main effect of locomotion | F_(1,19)_=0.69 | P=0.42 |
|  | Main effect of frequency | F_(1,19)_=13.22 | P=0.0018 |
|  | Locomotion x frequency interaction | F_(1,19)_=4.97 | P=0.038 |
| VH theta-  PL gamma | Main effect of locomotion | F_(1,11)_=0.93 | P=0.36 |
|  | Main effect of frequency | F_(1,11)_=4.97 | P=0.048 |
|  | Locomotion x frequency interaction | F_(1,11)_=0.86 | P=0.37 |
| VH theta-BLA gamma | Main effect of locomotion | F_(1,6)_=1.66 | P=0.24 |
|  | Main effect of frequency | F_(1,6)_=18.21 | P=0.0053 |
|  | Locomotion x frequency interaction | F_(1,6)_=2.29 | P=0.18 |
| PL theta-BLA gamma | Main effect of locomotion | F_(1,6)_=1.79 | P=0.23 |
|  | Main effect of frequency | F_(1,6)_=22.69 | P=0.0031 |
|  | Locomotion x frequency interaction | F_(1,6)_=1.84 | P=0.22 |
